# Intraindividual Reliability of Opportunistic Computed Tomography–Assessed Adiposity and Skeletal Muscle Among Breast Cancer Patients

**DOI:** 10.1093/jncics/pkac068

**Published:** 2022-10-12

**Authors:** Livingstone Aduse-Poku, Dheeraj R Gopireddy, Mauricio Hernandez, Chandana Lall, Joel Divaker, Sara M Falzarano, Shahla Masood, Susmita Datta, Weizhou Zhang, Ara Jo, Jiang Bian, Ting-Yuan David Cheng

**Affiliations:** Department of Epidemiology, College of Public Health and Health Professions and College of Medicine, University of Florida, Gainesville, FL, USA; Department of Radiology, College of Medicine–Jacksonville, University of Florida, Jacksonville, FL, USA; Department of Radiology, College of Medicine–Jacksonville, University of Florida, Jacksonville, FL, USA; Department of Radiology, College of Medicine–Jacksonville, University of Florida, Jacksonville, FL, USA; Department of Epidemiology, College of Public Health and Health Professions and College of Medicine, University of Florida, Gainesville, FL, USA; Department of Pathology, Immunology and Laboratory Medicine, College of Medicine, University of Florida, Gainesville, FL, USA; Department of Pathology and Laboratory Medicine, College of Medicine–Jacksonville, University of Florida, Jacksonville, FL, USA; Department of Biostatistics, College of Public Health and Health Professions and College of Medicine, University of Florida, Gainesville, FL, USA; Department of Pathology, Immunology and Laboratory Medicine, College of Medicine, University of Florida, Gainesville, FL, USA; Department of Health Services Research, Management and Policy, College of Public Health and Health Professions, University of Florida, Gainesville, FL, USA; Department of Health Outcomes and Biomedical Informatics, College of Medicine, University of Florida, Gainesville, FL, USA; Division of Cancer Prevention and Control, Department of Internal Medicine, College of Medicine, The Ohio State University, Columbus, OH, USA

## Abstract

**Background:**

Adiposity and skeletal muscle levels assessed on computed tomography (CT) scans are prognostic indicators for patients with breast cancer. However, the intraindividual reliability of temporal changes in body composition assessed on opportunistic CT scans is unclear.

**Methods:**

This retrospective study included 50 patients newly diagnosed with breast cancer who had archived CT scans pre- and postsurgery for breast cancer. The third lumbar CT image was segmented for areas of 3 types of adipose tissues and 5 different densities of skeletal muscles. Mean and percent changes in areas pre- vs postsurgery were compared using Wilcoxon signed rank tests. Intraclass correlation coefficients (ICCs) with 95% confidence intervals were assessed. A 2-sided *P* less than .05 was considered statistically significant.

**Results:**

Mean (SD) age at diagnosis was 58.3 (12.5) years, and the interval between CT scans was 590.6 (536.8) days. Areas for body composition components were unchanged except for intermuscular adipose tissue (mean change = 1.45 cm^2^, 6.74% increase, *P* = .008) and very high-density muscle (mean change = −0.37 cm^2^, 11.08% decrease, *P* = .01) during the interval. There was strong intraindividual reliability in adipose tissue and skeletal muscle areas on pre- vs postsurgery scans overall (ICC = 0.763-0.998) and for scans collected 3 or less years apart (ICC = 0.802-0.999; 42 patients).

**Conclusions:**

Although some body composition components may change after breast cancer surgery, CT scan assessments of body composition were reliable for a 3-year interval including the surgery. These findings inform measurement characteristics of body composition on opportunistic CT scans of patients undergoing surgery for breast cancer.

The impact of body composition on cancer outcomes is of great clinical importance. Accurate body composition measurement is essential in identifying patients with breast cancer who are at high risk of poor prognosis because this measurement distinguishes between adipose tissues and skeletal muscle components (1). Conventionally, body mass index (BMI), which is a composite measure of weight and height, has been used as a risk factor for prognosis, but BMI does not consider body composition in regard to actual tissue characterization, such as levels of adiposity and skeletal muscles (2). Body composition can be assessed using dual-energy radiograph absorptiometry (DEXA). However, DEXA cannot differentiate between various body fat segments, and its accuracy decreases for individuals with obesity compared with normal-weight individuals (3). Clinical computed tomography (CT) and magnetic resonance imaging scans are the most accurate and feasible methods to measure body composition. These methods can distinguish between visceral adipose tissue (VAT), subcutaneous adipose tissue (SAT), intermuscular adipose tissues, and various densities of skeletal muscles (4). In addition, compared with BMI, muscle and fat masses assessed using CT scans are more strongly associated with survival among patients with nonmetastatic breast cancer (5).

Epidemiologic studies using CT images for body composition assessment mainly rely on archived clinical scans (5,6). A major limitation of such an approach is that clinical CT images are obtained “opportunistically,” that is, for staging or for noncancer issues and are not actively collected. In general, patients with early-stage breast cancer (0 and I) are less likely than patients with an advanced-stage disease to receive a CT scan for staging. Body composition may change during the course of cancer treatment because of factors such as surgery (7-10). However, it is largely unknown whether opportunistically obtained CT-based measures of body composition represent true body composition over time. Understanding the reliability of body composition assessed on archived CT scans can be cost-saving because a CT or DEXA scan specifically for body composition assessment may not be needed.

The objective of this study was to assess the intraindividual reliability of CT-assessed body composition among patients who underwent surgery for the treatment of breast cancer to quantify the measurement characteristics of the method.

## Methods

### Participant Selection

Women who received breast cancer treatment at the University of Florida Health Shands Hospital from October 2011 to April 2020 were identified through the local tumor registry linked to an electronic medical record system. Eligible participants were women aged 20-75 years at diagnosis with  newly diagnosed nonmetastatic breast cancer, receiving a lumpectomy or mastectomy, and having an archived abdominal  or  pelvic CT scan, including positron emission tomography–CT scans. We excluded patients with a previous cancer diagnosis (except for nonmelanoma skin cancer), a history of diabetes, or pregnancy at the time of CT. We considered patients who had at least 2 scans: at least 1 collected before surgery and at least 1 collected after surgery. For patients with more than 2 scans obtained either before or after surgery, we prioritized scans obtained closest to surgery. In total, 50 patients were included in this study. The protocol was approved (IRB201800102) by the institutional review board at the University of Florida, which also waived the need to obtain informed patient consent.

### Clinical Data Collection

Sociodemographic variables, including race and ethnicity obtained through self-report, age at breast cancer diagnosis, weight, and height, were obtained from the electronic medical record system. When multiple weights and heights were available, the measurements closest to the time of breast cancer diagnosis were selected. Clinical and pathological variables, including cancer stage and grade, and estrogen receptor, progesterone receptor, and HER2 statuses, were provided by tumor registries and pathology reports. Variables associated with the cancer diagnosis, surgery, and chemotherapy were extracted using Current Procedural Terminology codes (Supplementary Table 1, available online).

### Body Composition Measurements

CT images were extracted using Current Procedural Terminology codes (74150, 74160, 74170, 74176, 74177, and 74178; Supplementary Table 1, available online). CT images were reviewed in Digital Imaging and Communications in Medicine format, and a single-slice image of the third lumbar vertebra (L3) was selected. The areas of skeletal muscles and adipose tissues based on L3 images are strongly correlated with whole-body volumes of skeletal muscles and adipose tissues (1). We excluded patients with CT images unsuitable for assessment because of lack of clarity, body images partially out of the image field, and heavy distortion. With the guidance of 2 board-certified, fellowship-trained abdominal radiologists, a single investigator analyzed the CT images using SliceOmatic version 5.0 revision 7 (TomoVision, Montreal, Canada). We used Hounsfield unit (HU) thresholds, representing the physical properties of tissues expressed in numerical form, to segment the various types of adipose and muscle tissues. A protocol for CT image annotation was developed using HU ranges, which were keyed into a script to aid in the semi-automatic segmentation and annotation of the L3 images. The area of total adipose tissue (TAT) comprised SAT, intermuscular adipose tissue, and VAT areas (11). SAT was selected by limiting the measurements to a lower attenuation of −190 HU, with an upper limit of −30 HU. Intermuscular adipose tissue and VAT were limited to HU ranges of −190 to −30 HU and −150 to −50 HU, respectively.

The skeletal muscle components of the body composition were classified based on their densities into very low-density muscles (VLDMs), low-density muscles (LDMs), normal-density muscles (NDMs), high-density muscles (HDMs), and very high-density muscles (VHDMs). VLDMs, LDMs, and NDMs were limited to the HU ranges of −29 to 0 HU, 0–35 HU, and 35–101 HU, respectively. The range for HDMs was 101–151 HU, and 151–200 HU was calibrated as the range for VHDMs. The total skeletal muscle area was calculated as the sum of the VLDM, LDM, NDM, HDM, and VHDM areas (4,12). We examined intermethod reliability by comparing our semiautomated method with a manual segmentation method annotating only SAT, VAT, and muscle using data from 273 patients with breast cancer with no replicated scans (13). Our results showed that annotated areas from the semiautomated method were highly correlated (correlation coefficient, *r *=* *0.936 for SAT, 0.945 for VAT, and 0.763 for muscle) with those from the manual segmentation method, but the former tended to provide smaller areas than the latter (Supplementary Table 2, available online).

The L3 CT images of patients with breast cancer were also used to measure waist circumference via the Snake function in SliceOmatic.

### Statistical Analysis

The distribution of the continuous variables was described by means and SDs or by medians, interquartile ranges, and minimum and maximum values. Categorical variables were described by frequencies and percentages. The mean changes in the areas of adipose tissue and skeletal muscle were assessed using Wilcoxon signed rank tests because the distributions for most components were skewed as assessed by Lilliefors-corrected Kolmogorov-Smirnov tests and Shapiro-Wilk normality tests.

To assess intraindividual reliability for body composition areas, we calculated intraclass correlation coefficients (ICCs), estimating the variance between patients divided by the sum of the variance between patients and within patients. Thus, an ICC close to 1 indicated high intraindividual reliability because most of the variance came from between patients, not between CT scans within a patient. The ICC estimates and their 95% confidence intervals were calculated using SPSS statistical package, version 24.0 (SPSS Inc, Chicago, IL, USA), based on a single-rating, consistency agreement, 2-way mixed-effects model (14). We used a mixed-effects model because, although the patients were randomly selected, the measurement of body composition on CT scans was considered a fixed effect because the selection of CT scans occurred based on the time of breast cancer surgery. The ICC estimate was considered the test-retest reliability because the rater effect, that is, the CT scan and our semiautomated method, was negligible. To assess change in the reproducibility of the CT images with time, we stratified the time intervals between the pre- and postsurgery CT scans into 3 or less years apart and longer than 3 years apart. A 2-sided *P* less than .05 was considered statistically significant.

## Results

For the entire patient population, the mean (SD) age, BMI, and waist circumference were 58.3 (12.5) years, 29.4 (7.0) kg/m^2^, and 103.5 (14.1) cm, respectively; 17.5% were Black patients, whereas 82.5% were White patients (Table 1). The majority of participants had stage I breast cancer (43.9%) with tumor grade II (52.8%). Fewer than one-half (43.6%) of the study participants underwent chemotherapy. The proportions of patients with positive estrogen receptor, progesterone receptor, or HER2 status were 79.5%, 71.1%, and 15.8%, respectively. The mean (SD) interval between presurgery scans and surgery was 276.5 (375.9) days and between surgery and postsurgery scans was 342.7 (331.9) days (Table 2). The mean (SD) interval between presurgery and postsurgery scans was 590.6 (536.8) days.

**Table 1.** Patient and clinical characteristics (N = 50)

Characteristics	No. (%)
Mean age at breast cancer diagnosis (SD), y	58.3 (12.5)
Mean body mass index (SD), kg/m^2^	29.4 (7.0)
Mean waist circumference on presurgery scan (SD), cm	103.5 (14.1)
Race and ethnicity	
Black	7 (17.5)
White	33 (82.5)
Missing	10
Cancer stage	
I	17 (43.9)
II	15 (38.2)
III	7 (17.9)
Missing	11
Tumor grade	
I	5 (13.9)
II	19 (52.8)
III	12 (33.3)
Missing	14
Chemotherapy	
No	22 (56.4)
Yes	17 (43.6)
Missing	11
Estrogen receptor status	
Negative	8 (20.5)
Positive	31 (79.5)
Missing	11
Progesterone receptor status	
Negative	11 (28.9)
Positive	27 (71.1)
Missing	12
HER2 status	
Negative	30 (78.9)
Positive	6 (15.8)
Equivocal	2 (5.3)
Missing	12

**Table 2.** Summary statistics of the intervals of computed tomography scans

Intervals	Mean (SD)	Minimum	Quartile 1	Median	Quartile 3	Maximum
Between pre- and postsurgery scans, d	590.6 (536.8)	22	209	438	760	2132
Between presurgery scan and surgery, d	276.5 (375.9)	1	28	219	413	1776
Between surgery and postsurgery scan, d	342.7 (331.9)	17	107	129	544	1316

Comparing body composition components on CT scans obtained before and after surgery (Table 3), both TAT and total skeletal muscle appeared to be decreased, although the changes were not statistically significant. Specific body composition components were also unchanged except for intermuscular adipose tissue (mean change = 1.45 cm^2^, 6.74% increase, *P* = .008) and VHDM (mean change −0.37 cm^2^, 11.08% decrease, *P* = .01).

**Table 3.** Adipose and muscle tissue areas before vs after surgery for 50 patients with breast cancer

Body composition component	Presurgery area, mean (SD), cm^2^	Postsurgery area, mean (SD), cm^2^	Difference,^a^ mean (SD), cm^2^	Percent change,^b^ %	*P* ^c^
Subcutaneous adipose tissue	308.58 (165.41)	272.41 (160.19)	−36.17 (113.43)	−11.72	.17
Intermuscular adipose tissue	21.51 (76.16)	22.95 (73.80)	1.45 (5.82)	6.74	.008
Visceral adipose tissues	118.20 (75.79)	107.29 (78.32)	−10.91 (43.27)	−9.23	.27
Very low-density muscle	18.30 (16.32)	19.38 (20.45)	1.09 (6.52)	5.96	.65
Low-density muscle	34.89 (11.07)	33.30 (10.53)	−1.59 (8.90)	−4.56	.22
Normal-density muscle	62.85 (19.82)	62.11 (20.47)	−0.74 (17.73)	−1.17	.63
High-density muscle	6.01 (11.25)	6.03 (13.06)	0.03 (2.89)	0.50	.77
Very high-density muscle	3.34 (1.45)	2.97 (1.19)	−0.37 (0.99)	−11.08	.01
Total adipose tissue	448.29 (202.07)	402.66 (209.24)	−45.64 (146.05)	−10.18	.19
Total skeletal muscle	125.38 (28.47)	123.79 (29.43)	−1.59 (17.13)	−1.27	.38
Waist circumference	103.53 (14.06)^d^	104.33 (13.62)^d^	0.80 (12.51)^d^	0.01	.65

a Difference estimated as postsurgical area minus presurgical area.

b Percent change = $\left(\frac{\mathrm{Postsurgery}\;\mathrm{area}\;-\;\mathrm{presurgery}\;\mathrm{area}}{\mathrm{Presurgery}\;\mathrm{area}}\right)\times 100$.

c *P* values are from Wilcoxon signed rank tests.

d In centimeters.

The ICC values for pre- and postsurgery adipose tissue and skeletal muscle areas ranged from 0.763 to 0.998 (Table 4), indicating high intraindividual reliability and thus strong similarity in the areas measured for each tissue type before and after surgery. After stratifying CT scans collected before and after surgery by those obtained 3 or less years apart and longer than 3 years apart (Table 5), we found that the ICC values for areas of adipose tissues and for skeletal muscles indicated a high degree of reproducibility between scans collected 3 or less years apart (ICC = 0.802-0.999) but attenuated reproducibility for scans taken longer than 3 years apart (ICC = −0.055-0.738).

**Table 4.** Intraindividual reliability of adipose and muscle areas measured on CT scans collected before vs after surgery, assessed by ICCs and 95% confidence intervals^a^

Body composition component	ICC (95% CI)
Subcutaneous adipose tissue	0.852 (0.580 to 0.922)
Intermuscular adipose tissue	0.998 (0.997 to 0.999)
Visceral adipose tissues	0.911 (0.842 to 0.950)
Very low-density muscle	0.968 (0.943 to 0.982)
Low density muscle	0.794 (0.638 to 0.882)
Normal-density muscle	0.763 (0.582 to 0.866)
High density muscle	0.986 (0.975 to 0.992)
Very high-density muscle	0.823 (0.673 to 0.903)
Total adipose tissue	0.846 (0.724 to 0.914)
Total skeletal muscle	0.905 (0.833 to 0.946)

a CT = computed tomography; ICC = intraclass correlation coefficient.

**Table 5.** Reproducibility of body composition areas measured on pre- and postsurgery CT scans collected 3 or less than 3 years vs more than 3 years apart, assessed by ICCs and 95% confidence intervals^a^

Body composition	≤3 y apart (N = 42)	>3 y apart (N = 8)
	ICC (95% CI)	ICC (95% CI)
Subcutaneous adipose tissue	0.945 (0.899 to 0.971)	0.355 (−0.848 to 0.849)
Intermuscular adipose tissue	0.999 (0.997 to 0.999)	0.525 (−1.851 to 0.908)
Visceral adipose tissue	0.940 (0.889 to 0.968)	0.684 (−0.205 to 0.933)
Very low-density muscle	0.968 (0.941 to 0.983)	0.738 (−0.097 to 0.945)
Low-density muscle	0.802 (0.633 to 0.893)	0.610 (−0.859 to 0.921)
Normal-density muscle	0.818 (0.660 to 0.902)	0.085 (−7.810 to 0.833)
High-density muscle	0.986 (0.974 to 0.993)	0.730 (−0.249 to 0.945)
Very high-density muscle	0.860 (0.719 to 0.928)	0.272 (−3.473 to 0.860)
Total adipose tissue	0.931 (0.872 to 0.963)	0.402 (−0.675 to 0.858)
Total skeletal muscle	0.937 (0.883 to 0.966)	−0.055 (−5.400 to 0.796)

a CI = confidence interval; CT = computed tomography; ICC = intraclass correlation coefficient.

## Discussion

This retrospective study was, to our knowledge, the first study to examine the intraindividual reliability of the areas of various adipose tissues and skeletal muscles measured on archived CT scans collected from patients who underwent surgery for the treatment of breast cancer. We found a strong intraindividual reliability in the measures of the areas of the adipose tissues and skeletal muscle when the CT scans were obtained within 3 years of each other, but the reliability was attenuated for scans obtained longer than 3 years apart. The attenuation may be due to the small number of patient pairs (N = 8). This finding adds to the literature on the measurement characteristics of CT-assessed body composition. Other strengths of using CT-assessed body composition are its high intraobserver and interobserver reliability. For example, a study examining the reproducibility and repeatability of CT-based measurements of abdominal adipose tissues in patients with obesity found high interrater reliability for both VAT (coefficient of variation range = 1.08%-2.13%) and SAT (coefficient of variation range = 0.47%-1.16%) (15). In addition, in another reliability study, VAT was seen to have high intrainvestigator reliability, with coefficients of variation ranging from 0.2% to 3.4% and *R*^2^ = 0.99 (16).

Changes in areas of adipose tissues after surgeries may be due to substantial changes in diet and physical activity levels (6,7,17) as well as concomitant cancer treatments, such as chemotherapy and radiation therapy (18-22). We did not observe a statistically significant change in the areas of VAT, SAT, or TAT, although intermuscular adipose tissue was increased during the scan interval involving surgery for treatment of breast cancer. However, studies assessing changes in adipose tissue areas after surgery have reported conflicting results. A study of 96 patients who underwent surgical resection for treatment of gastric cancer showed a statistically significant increase in VAT, SAT, and TAT after surgery (23). Conversely, in a retrospective cohort study, patients with colorectal cancer experienced a statistically significant decrease in SAT and VAT after colectomy (7). The discrepancies in the changes of adipose tissue between studies may be due to the variability in types of cancer, surgeries, or specific types of adipose tissue. Increases in adipose tissue for patients with cancer are concerning because adipocytes are thought to produce estrogen, adipokine, and inflammatory factors that enhance tumor progression and metastasis by reprogramming the metabolism of cancer cells (24). Intermuscular adipose tissue has been linked to decreased insulin sensitivity. Compared with VAT and SAT, intermuscular adipose muscle secretes more cytokines and chemokines (25). Whether the increase in intermuscular adipose tissue has clinical implications is unclear. Only 1 study reported a change in adipose tissue after surgery in association with risk of metastasis among patients with breast cancer (10). However, in that study, adipose tissue was measured by DEXA scan, and specific adipose tissue components could not be quantified.

A loss of muscle mass in patients with breast cancer is associated with functional decline, disability, and increased mortality (5,26,27). In this study, we found a statistically significant decrease in VHDM after surgery. This finding was similar to that of a prospective cohort study by Yoshida and colleagues (28), who found that the skeletal muscle mass index of 71 patients decreased statistically significantly 2 years after esophagectomy. This observation may be due to lack of physical exercise or surgical stress, which is known to accelerate lipolysis and proteolysis, leading to the consumption of muscle proteins (29). Research on biomarkers, such as lipokines and cytokines, may help us understand the origins and underlying mechanisms for the area changes in adipose tissue and skeletal muscle.

We used semiautomated, high-resolution segmentation methods to differentiate between various adipose tissues and 5 levels of skeletal muscle (from very high density to very low density). CT is typically considered the gold standard for measuring body composition owing to its ability to accurately differentiate between adipose tissue and skeletal muscle components (30). It was evident in our study that changes in specific body composition components were observed despite waist circumference—a surrogate of abdominal fat and VAT—remaining unchanged during the scan intervals. However, the limitations of using CT scans to measure body composition include cost and availability. We have provided evidence that CT scans obtained up to 3 years apart are highly correlated with each other, even after surgery. Therefore, a preoperative CT scan prescribed for cancer staging in patients with breast cancer may be readily used for assessing body composition and indicating body composition after diagnosis or treatment. In addition, for patients with breast cancer who are not prescribed a CT scan for cancer staging, an archived CT scan taken for other indications within a few years before diagnosis or surgery may be used to indicate body composition. Assessing body composition is important for patients with cancer because it provides information for prognosis as well as for prescribing physical activity or weight management programs.

Our findings may also increase the research utility of using opportunistic CT scans to assess body composition. For example, a study can broaden its inclusion criteria to patients with breast cancer with early-stage (0 or I) disease who may have an archived CT scan. In addition, a longer time window between a CT scan and breast cancer diagnosis or surgery can be applied as part of the inclusion criteria. These less restricted criteria would substantially increase the generalizability of research.

The limitations of this study included its limited generalizability for patients with breast cancer who did not undergo mastectomy and a relatively small sample size. CT scans were not collected during mastectomy; thus, changes in body composition may not be related to surgery. In addition, the measurement of adipose tissues and skeletal muscle areas using L3 CT scans may be less accurate than volumetric analysis, which was unavailable for this study. However, single-slice abdominal cross-sectional areas obtained at the L3 vertebra are strongly correlated with whole-body volumes of adipose tissue and skeletal muscle (1).

In conclusion, this retrospective study found that body composition as assessed by opportunistic CT images was reliable for a 3-year period among patients who underwent surgery for treatment of breast cancer. These findings inform the clinical utility and measurement characteristics of body composition using opportunistic CT scans. Further research is needed to examine the impact of body composition changes on breast cancer outcomes.

## Funding

Research reported in this publication was supported by the National Institutes of Health (NIH) National Cancer Institute awards K07CA201334 and R37CA248371 and the University of Florida Clinical and Translational Science Institute, which is supported in part by the NIH National Center for Advancing Translational Sciences under award number UL1 TR001427. W.Z. was partially supported by NIH grants CA200673 (W.Z.), CA203834 (W.Z.), CA260239 (W.Z.), DOD/CDMRP grants BC180227 and BC200100 (W.Z.). W.Z. was also supported by an endowment fund from the Dr and Mrs James Robert Spenser Family.

## Notes

**Role of the funder:** The funders had no role in the design and conduct of the study; the collection, management, analysis, or interpretation of the data; preparation, review, or approval of the manuscript; or the decision to submit the manuscript for publication.

**Disclosures:** All authors report no conflicts of interest. T.-Y.D.C., a *JNCI Cancer Spectrum* Deputy Editor and co-author on this manuscript, was not involved in the editorial review or decision to publish the article.

**Author contributions:** Conceptualization: LAP, TYDC. Data curation: LAP, JB, JD. Formal analysis: LAP. Investigation: LAP, TYDC. Resources: TYDC, JB, DRG, MH, CL, SMF, SM, WZ, SD, AJ. Funding acquisition: TYDC. Writing–original draft: LAP, TYDC. Writing–review and editing: all.

**Disclaimer:** The content is solely the responsibility of the authors and does not necessarily represent the official views of the National Institutes of Health.

**Acknowledgements:** We thank the Integrated Data Repository team, part of the Clinical and Translational Institute at the University of Florida, for assisting with this project.

**Prior presentations:** The abstract of this article was presented at the 2022 AACR Annual Meeting (April 8-13, 2022, New Orleans, LA, USA).

## Supplementary Material

pkac068_Supplementary_Data

## Data Availability

The data underlying this article will not be available to the public to protect patient privacy. The data generated by the analysis are available in the article and its supplementary materials.
