# Supplementary material for: Intraindividual Reliability of Opportunistic Computed Tomography–Assessed Adiposity and Skeletal Muscle Among Breast Cancer Patients
Source: JNCI Cancer Spectr. 2022 Oct 12;6(6):pkac068. doi: 10.1093/jncics/pkac068 (PMC9623424; doi:10.1093/jncics/pkac068)
Supplement: pkac068_Supplementary_Data [file pkac068_supplementary_data.pdf]

Supplementary Material

Supplementary Table 1. *International Classification of Diseases* (*ICD)* and *Current Procedural Terminology* (*CPT)* codes for breast cancer case identification and procedures

Variable	*CPT* Codes	*ICD-9* Diagnosis or Procedure Codes	*ICD-10* Diagnosis or Procedure Codes
A. Breast cancer diagnosis	-	174	C50 (and C50 for ICD-O-3)<br>Z17.0 for ER+ tumors and Z17.1 for ER- tumors
B. Computed tomography scan	74150, 74160, 74170, 74176, 74177, 74178	-	-
C. Breast cancer treatment			
Lumpectomy	19120, 19125, 19126, 19160, 19162, 19301, 19302	85.2, 85.20-85.25	0HBT, 0HBU, 0HBV, 0HPT, 0HPU, 0HTT, 0HTU, 0HTV
Mastectomy	19180,19182,19200,19220,19240, 19303,19304, 19305,19306, 19307	85.33-85.48, including 85.4	
Any radiation	77371-77373, 77401-77416, 77418, 77422-77425, 77520-77525, 77761-77789, G0174, G0251, G0339, G0340, 0182T	92.2, 92.20-92.27, 92.29, 92.3, 92.30-92.39, 92.4, 92.41	
External beam radiation (whole-breast irradiation or post-mastectomy radiation therapy)	77401-77416, 77418, G0174	92.21 - 92.26	
Brachytherapy	77785, 77786, 77787		
Axillary surgery	38500, 38525, 38740, 38745, 19162, 19200, 19220, 19240, 19302, 19305, 19306, 19307	40.23, 40.3, 40.51, 85.43, 85.44, 85.45, 85.46, 85.47, 85.48	
Chemotherapy	96400-96549, J9000-J9999 (excluding: J9003, J9165, J9175, J9202, J9209, J9212-J9226, J9240, J9395), Q0083-Q0085, J8510, J8515, J8520, J8521, J8530, J8560, J8561, J8562, J8565, J8600, J8610, J8650, J8700, J8705, J8999, G0355, G0359, G0360, G0361, G0362	99.25	V58.1, V66.2, V67.2

Supplementary Table 2. Intermethod reliability of adipose tissues and skeletal muscle area measurements (N= 273)

Body composition component	Bias (X_1−X_2), cm^2	*R*
Subcutaneous adipose tissue	27.06	0.936
Visceral adipose tissue	19.11	0.945
Muscle[a]	10.70	0.763

X_1 = manual segmentation.

X_2 = semi-automated segmentation.

[a] Compared with total skeletal muscle in X_2.
